# Achieving low-emissivity materials with high transmission for broadband radio-frequency signals

**DOI:** 10.1038/s41598-017-04988-9

**Published:** 2017-07-07

**Authors:** Liu Liu, Huiting Chang, Tao Xu, Yanan Song, Chi Zhang, Zhi Hong Hang, Xinhua Hu

**Affiliations:** 1 Department of Materials Science, Key Laboratory of Micro- and Nano-Photonic Structures (Ministry of Education), and Laboratory of Advanced Materials, Fudan University, Shanghai, 200433 China; 2 College of Physics, Optoelectronics and Energy & Collaborative Innovation Center of Suzhou Nano Science and Technology, Soochow University, Suzhou, 215006 China

## Abstract

The use of low-emissivity (low-e) materials in modern buildings is an extremely efficient way to save energy. However, such materials are coated by metallic films, which can strongly block radio-frequency signals and prevent indoor-outdoor wireless communication. Here, we demonstrate that, when specially-designed metallic metasurfaces are covered on them, the low-e materials can remain low emissivity for thermal radiation and allow very high transmission for a broad band of radio-frequency signals. It is found that the application of air-connected metasurfaces with subwavelength periods is critical to the observed high transmission. Such effects disappear if periods are comparable to wavelengths or metal-connected structures are utilized. The conclusion is supported by both simulations and experiments. Advantages such as easy to process, low cost, large-area fabrication and design versatility of the metasurface make it a promising candidate to solve the indoor outdoor communication problem.

## Introduction

Reducing energy consumption and improving energy efficiency are important issues for humans in the 21st century^1–5^. Among the global energy consumption, a considerable proportion (*e.g*. 12.3% in U.S.) is consumed in heating and cooling for buildings^5^. For energy saving, an efficient method is to apply low-emissivity (low-e) materials at the surfaces of buildings^6–9^. For instance, aluminum foil phenolic foam boards and low-e glass can be mounted on the outer walls of buildings and windows, respectively. Hence, a major way of heat exchange (namely thermal radiation) between buildings and environment can be inhibited. As a result, less power can be consumed to maintain optimum temperature in buildings.

On the other hand, some wireless appliances, such as cell phones, have become indispensable in modern life. However, the use of such telecommunication equipments can be strongly influenced by low-e materials in buildings, because such materials are coated with metallic films and allow very low transmission for radio-frequency (RF) signals^10^. Although some schemes have been proposed to solve the problem, they have their own limitations. For example, frequency selective windows work only in narrow bands^10, 11^, while external antennas and femtocells bring challenges for installers or users.

Metasurfaces are a class of artificial sheet materials with subwavelength thickness that could modulate the behaviors of reflected and transmitted electromagnetic (EM) waves by arranging periodic patterns on the surface of materials^12, 13^. In recent years, metasurfaces have drawn increasing attention due to their promising applications, such as planar lens, vortex generator, beam deflector, perfect absorbers, polarization converters and so on^12–20^.

In this paper, we show that the introduction of metasurfaces can greatly improve the transmission of microwaves through low-e materials. By theoretically simulating the influence of certain geometry patterns, namely air-connected and metal-connected structures, and the periodicity on transmission, we found that the air-connected structure can reach a very high transmission (>90%) for a wide band (0–6 GHz) of RF signals. Experiments were also conducted to verify the simulation results. Furthermore, advantages such as easy to process, low cost, large-area fabrication and design versatility of the metasurface make it a promising candidate to solve the indoor outdoor communication problem.

## Results

### Ordinary low-e materials

**Figure 1:**
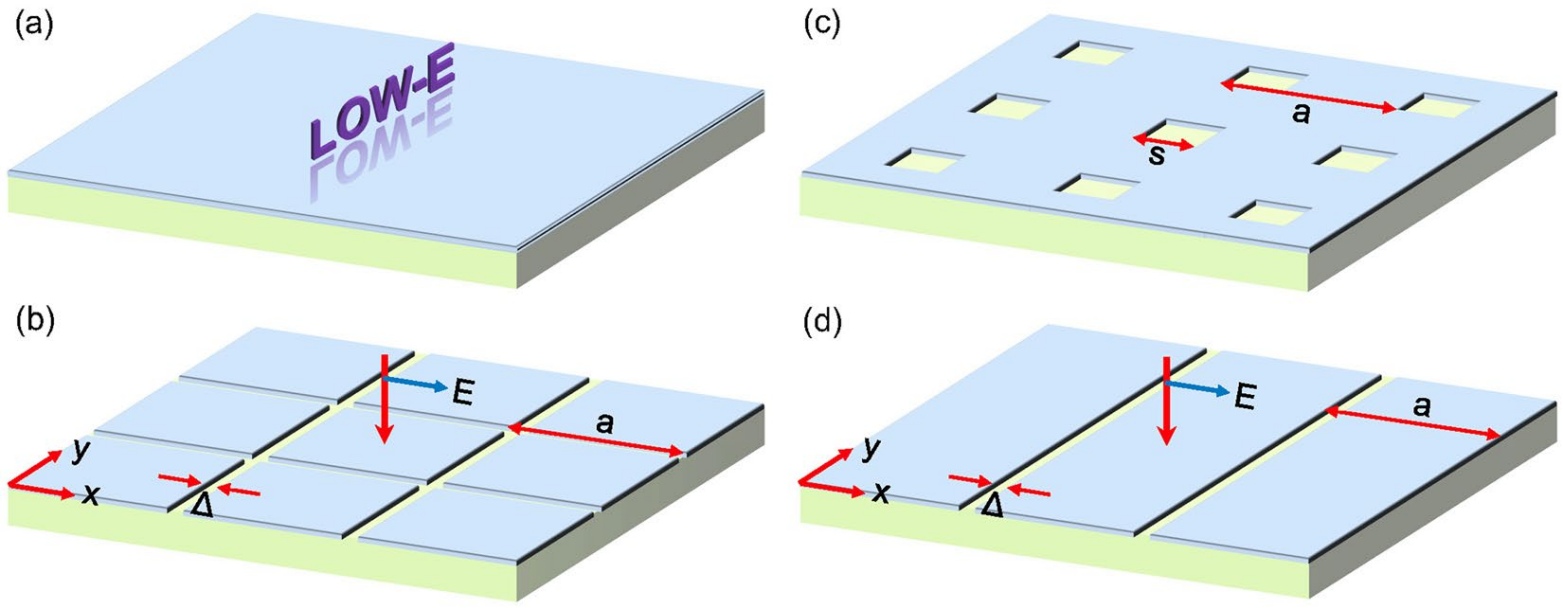
Redesign of low-e materials by using metasurfaces. (**a**) Schematic of ordinary low-e materials that is a substrate covered by a metallic film. (**b**,**c**) The same as (**a**) but with the metallic film designed to be of an air-connected and metal-connected pattern, respectively, in which *a* is the periodicity, Δ is the width of air slit, and *s* is the size of the air hole. The thicknesses of the metallic coating and the substrate are *t*_1_ and *t*_2_, respectively. (**d**) The same as (**b**) but with the metal patches replaced by metal stripes. The structures in (**b**,**d**) have almost the same transmission for normal incidence with the *E*-field in the *x* direction.

A low-e material consists of a dielectric layer coated by a metallic film, as schematically shown in Fig. 1(a). Common low-e materials include low-e glass and aluminum foil phenolic foam boards where 8–18 nm-thick-Ag films and 6–200 *μ*m-thick Al foils are utilized, respectively. The two kinds of low-e materials can be mounted on the windows and outer walls of buildings, respectively.

**Figure 2:**
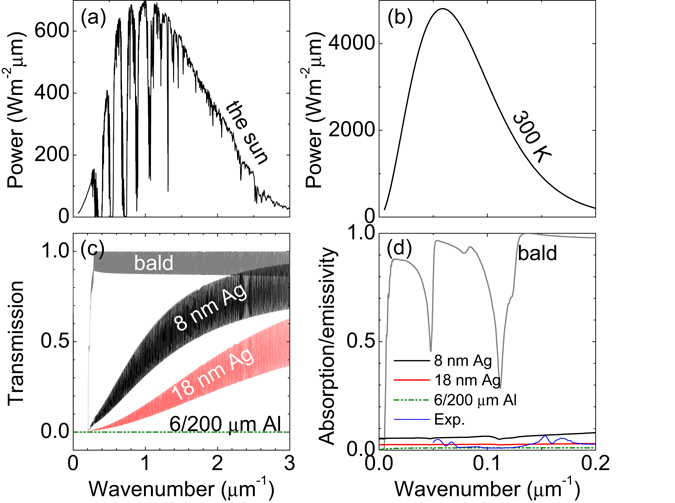
Optical and infrared properties of common low-e materials. (**a**) The air mass 1.5 direct normal and circumsolar spectrum. (**b**) Radiant power of the surface of a blackbody at temperature *T* = 300 K. (**c**) Calculated transmission and (**d**) absorption/emissivity of five pieces of 1 mm-thick glass that are bald (grey line), 8 nm-thick-Ag coated (black line), 18 nm-thick-Ag coated (red line), and 6 or 200 *μ*m-thick-Al covered (green lines), respectively. The blue line in (**d**) is the measured spectrum for a piece of 1 mm-thick glass coated by 3-nm-thick TiO_2_, 1-nm-thick Au, 12-nm-thick Ag, 1-nm-thick Au and 100-nm-thick TiO_2_.

Figure 2(c) shows the transmission spectra for the two kinds of low-e materials. As shown in the air mass 1.5 direct normal and circumsolar spectrum^21^, the radiation of sun is mainly in the wavenumber range between 0.1 to 3 *μ*m^−1^, where the wavenumber of visible light is from 1.28 to 2.56 *μ*m^−1^ [see Fig. 2(a)]. It can be seen that the Al foil phenonic foam board is opaque in all the infrared and visible range, because the covered metallic layers are very thick and can strongly reflect EM waves [see Fig. 2(c)]. Similarly, low-e glass is also opaque in the infrared range with wavenumber lower than 0.2 *μ*m^−1^. But since thin metallic coatings are applied, the low-e glass is semi-transparent in the visible range. At wavelength of 550 nm (or wavenumber of 1.82 *μ*m^−1^), the transmission is 68% (29%) for 8 (18) nm-thick-Ag-coated glass, which is lower than that (95%) of common glass.

The thermal radiant power from a surface with area *A* is given by $P=A\pi {\int }_{0}^{\pi /2}d\theta \,\sin \,2\theta {\int }_{0}^{\infty }d\lambda {I}_{BB}(T,\lambda )\varepsilon (\lambda ,\theta )$, where *ε*(*λ*, *θ*) = *α*(*λ*, *θ*), *ε*(*λ*, *θ*) and *α*(*λ*, *θ*) are the emissivity and absorptivity of the surface, respectively^2^. ${I}_{BB}(T,\lambda )=2h{c}^{3}{\lambda }^{-5}/[{e}^{hc/(\lambda {k}_{B}T)}-\mathrm{1]}$ is the spectral radiance of a blackbody at temperature *T*, where *h* is the Plank’s constant, *k*_*B*_ is the Boltzmann constant, *c* is the speed of light and *λ* is the wavelength. For a blackbody at room temperature (*T* = 300 *K*), the thermal radiation occurs mainly in the wavenumber range between 0.02 to 0.2 *μ*m^−1^ [see Fig. 2(b)]. Hence, the thermal radiation from bodies at room temperature inside a building cannot transmit through the walls and window glass of the building [see Fig. 2(c)]. However, the surfaces of a building such as the walls and window glass can radiate heat toward environments [see the gray line in Fig. 2(d)]. Such thermal radiation can be inhibited by applying low-e materials at the surface of building.

Figure 2(d) shows the emissivity of the two low-e materials above mentioned. We can see that although the emissivity is large (85% at average) for common glass, it can be reduced to 6% (2%) when the glass is coated by a 8 (18) nm-thick Ag film. Lower emissivity (1%) can be achieved by using thicker (6–200 *μ*m) Al foils. Hence, when the low-e materials are used at the surfaces of buildings, the thermal exchange (due to heat radiation) between buildings and environments can be greatly inhibited.

**Figure 3:**
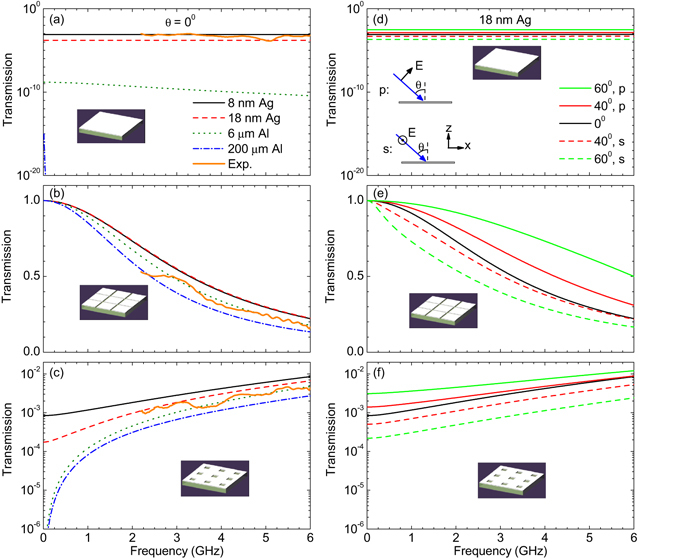
Making low-e materials transparent in GHz band by metasurfaces. (**a**–**c**) Calculated transmission spectra for the structures in Fig. 1(a–c) at normal incidence, respectively. The substrate has a thickness *t*_2_ = 1 mm and dielectric constant *ε* = 4. In (**b**,**c**), the periodicity *a* = 2 cm and the filling ratio of air is 0.1 in the coatings. The orange solid lines are the measured spectra for 1 mm-thick glass samples coated by 3-nm-thick TiO_2_, 1-nm-thick Au, 12-nm-thick Ag, 1-nm-thick Au and 100-nm-thick TiO_2_. (**d**–**f**) Calculated transmission spectra for the structures in Fig. 1(a–c) at oblique incidence, respectively. The substrate is coated by a 18 nm-thick Ag film and other parameters are the same as in (**a**–**c**).

However, due to the introduction of metallic coatings, the low-e materials can influence the applications of some wireless appliances, such as cell phones. Common third-generation (3 G) and fourth-generation (4 G) networks operate in the frequencies of 1.9–2.1 GHz and 2.3–2.6 GHz, respectively. In such frequency ranges, the transmission is only 10^−3^ (10^−4^) for low-e glass with 8 (18) nm-thick-Ag coating, while lower transmission of 10^−9^ (<10^−30^) exists for 6 (200) *μ*m-thick-Al covered composite board [see Fig. 3(a)].

### Novel low-e metamaterials

In order to solve this problem, we introduce the concept of metasurface to redesign the metallic films on low-e materials. We study two kinds of metasurfaces that are metallic films which are perforated by periodic holes and air grids, respectively, and covered on dielectric substrate [see Fig. 1(b,c)]. The former can also be viewed as an array of square metal patches. The space between metal patches is Δ and the size of square air holes is *s*. The metal patches and air holes are arranged in a 2D square lattice with primitive vectors along the *x* and *y* directions. The lattice periods are much larger than infrared wavelengths (*i.e. a* > 100 *μ*m), so that the system can be dealt with geometrical optics^22^. Hence, the absorption/emissivity of the metasurfaces can be estimated by the area average of absorption/emissivity, namely *ε* = *ε*_*m*_(1 − *f*_*air*_) + *ε*_*sub*_ *f*_*air*_, where *ε*_*m*_ ≈ 0 and *ε*_*sub*_ < 1 are the emissivity of metallic film and substrate, respectively, and *f*_*air*_ is the filling ratio of air in metasurfaces. When the filling ratios of air are small (*f*_*air*_ < 10%), the emissivity of metasurfaces can also be low (*ε* < 0.1). In the followings, we focus on the cases with *f*_*air*_ = 10%.

The wavelength is 12 cm for microwaves at 2.5 GHz (belonging to the 4 G band). We first consider the case of normal incidence and with *E*-field in the *x* direction. When the wavelength is much larger than the period, the metal patch array possesses almost the same transmission as the metal stripe array as shown in Fig. 1(d). Here, the metal stripes have a thickness *t*_1_, period *a* and interspace Δ. According to an effective medium theory^23^, such a metal stripe array (and the metal patch array) can be effectively viewed as a dielectric film with refractive index *n* = *a*/Δ and thickness ${t}_{1}^{^{\prime} }={t}_{1}/n$. For such a metallic metasurface in air, the transmission *T* = |4*nC*/[(1 + *n*)^2^ − (1 − *n*)^2^ *C* ^2^]|^2^ at normal incidence, where $C=\exp (i{k}_{0}{t}_{1})$ and *k*_0_ = 2*π*/*λ* is the wavenumber in vacuum. For a very thin film ($t\ll \lambda$), high transmission can thus be achieved (*T* ≈ 1). When the wavelength is much shorter than the period, geometric optics can be used to estimate the transmission of the metallic patch array, giving rise to moderate transmission (*T* ≈ *f*_*air*_).

For wavelengths much larger than the period, the above metallic metasurface can remain unity transmission (*T*′ = 1) if a larger filling ratio of air is applied (*f*_*air*_ = 0.9). According to the Babinet principle^24^, its complementary structure, namely a metallic film perforated by periodic holes with *f*_*air*_ = 0.1, thus possesses neglectable transmission (*T* ≈ 1 − *T*′ = 0). For wavelengths much shorter than the period, the transmission of a metallic film with periodic holes can also be estimated by geometric optics, namely *T* ≈ *f*_*air*_.

### Scattering-matrix simulations

To verify the above theory, we apply a scattering-matrix method (SMM) to simulate the two metallic metasurfaces^25–27^. 289 plane waves are applied in the SMM simulations. We first study the cases where the period *a* = 2 cm, and the substrate has a thickness *t*_2_ = 0.1 mm and dielectric constant *ε* = 4. Figure 3(b) shows the transmission for a metasurface with air-connected structure. It can be seen that the transmission is almost unity (*T* ≈ 1) at low frequencies, agreeing with above prediction. The transmission decreases with increasing frequencies and drops a little with increasing the thickness *t*_1_ of metallic film. At the frequency region of 4 G network (around 2.5 GHz), the transmission the metasurface is about 64% (64%) and 56% (48%) if an 8 (18) nm-thick Ag film and 6 (200) *μ*m-thick Al foil are applied, respectively. This corresponds to enhancement of 28 (38) and 88 (>300) dB, respectively, compared with the transmission of metallic films without structures.

Figure 3(c) illustrates the transmission of a metasurface with metal-connected structure. At 2.5 GHz, the transmission is found to be 0.2% (0.1%) and 0.07% (0.05%) if an 8 (18) nm-thick Ag film and 6 (200) *μ*m-thick Al foil are applied, respectively. Compared with metallic films without structures, the metasurfaces have enhanced transmission, while the absolute values remain small (*T* < 0.2%).

**Figure 4:**
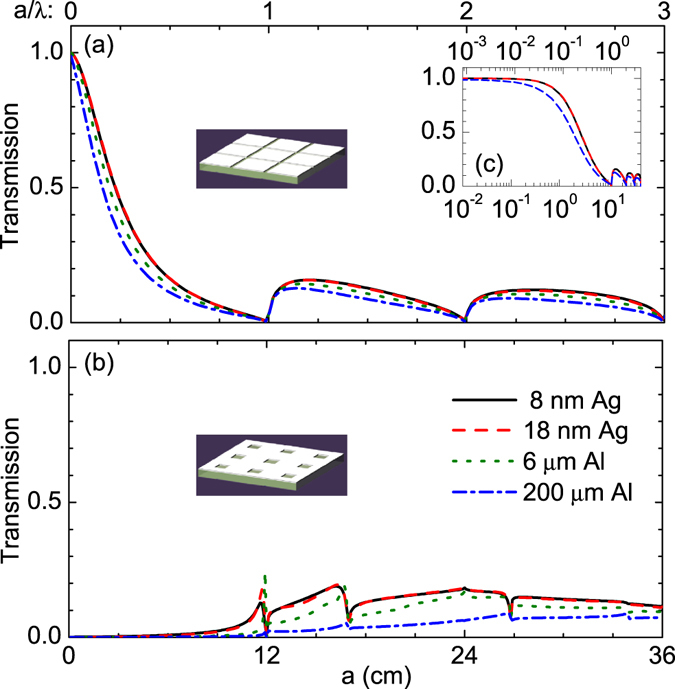
Transmission properties of low-e metasurfaces at 2.5 GHz. (**a**,**b**) Calculated transmission of the structure in Fig. 3(b,c) with different periodicity at frequency of 2.5 GHz, respectively. (**c**) Replotting of (**a**) with the periodicity *a* in a logarithmic scale.

Figure 4 shows the dependence of the transmission of metasurfaces on the period for incident waves at 2.5 GHz. For air-connected structures, the transmission is close to unity when the reduced frequency *a*/*λ* < 0.01 [see Fig. 4(c)]. When 0.01 < *a*/*λ* < 1, the transmission decreases with increasing period *a* [see Fig. 4(a)]. When *a*/*λ* = 1, the transmission is zero and the incident waves are strongly reflected by the metasurface. Such an effect is usually called Wood anormaly and can occur at *a*/*λ* = *j* with *j* being a positive integer^28, 29^. Between two neighbored transmission minimums (*j* < *a*/*λ* < *j* + 1), the transmission is always lower than *T*_*max*,*j*_. *T*_*max*,*j*_ decreases with increasing *j* and approaches the filling ratio of air (*f*_*air*_ = 0.1) for large *j*.

For metal-connected structures, the transmission is close to zero when *a*/*λ* < 0.5 [see Fig. 4(b)]. When *a*/*λ* > 0.5, the effect of Wood anormaly can be observed at more frequencies compared with the air-connected structures. Here, the transmission can be close to zero when $a/\lambda =\sqrt{{j}^{2}+{l}^{2}}$ (such as *a*/*λ* = 0, 1, $\sqrt{2}$, $\sqrt{5}\ldots$) with *j*, *l* = 0, 1, 2 … Between two neighbored transmission minimums (*e.g*. 0 < *a*/*λ* < 1), a transmission maximum exists which decreases with increasing frequencies and approaches the filling ratio of air (*f*_*air*_ = 0.1) for large frequencies. We note that similar to Fig. 3(b,c), the transmission in Fig. 4(a,b) decreases a little with increasing the thickness of metallic film.

The above results are for the case of normal incidence. In Fig. 3(d–f), we show more results for oblique incidence. Here, we focus on the samples with 18 nm-thick Ag coatings. The incident wavevector is in the *x*-*z* plane. The *E*-field of incident wave is either in the *x*-*z* plane (*i*.*e*. *p* polarization) or in the *y* direction (*i*.*e*. *s* polarization). Since the *E*-field should be in the *z* direction at the metal surface, the transmission at oblique incidence is higher (lower) than that at normal incidence for *p* (*s*) polarization. For incidence of natural polarized waves, the transmission at oblique incidence is close to that at normal incidence. Hence, the metasurfaces with metal patches still possess high transmission at oblique incidence. In contrast, the transmission remains very low for metal-connected metasurfaces at oblique incidence.

### Experiments

To verify the above theory, we prepared 1 mm-thick glass coated by 3-nm-thick TiO_2_, 1-nm-thick Au, 12-nm-thick Ag, 1-nm-thick Au and 100-nm-thick TiO_2_. The measured infrared absorption/emissivity spectrum is plotted as the blue solid line in Fig. 2(d). The average infrared emissivity is found to be about 3%, agreeing with the calculated value. The microwave transmission spectra are also measured as shown as the orange solid lines in Fig. 3(a–c). When the metallic coating is not patterned, the transmission is between 10^−3^ and 10^−4^ for 2–6 GHz [Fig. 3(a)]. Low transmission remains when the metallic layer is perforated by a square hole array with *a* = 2 cm and *s* = 6.3 mm [Fig. 3(c)]. In contrast, when an air grid with *a* = 2 cm and Δ = 1.0 mm is applied in the metallic coating, the transmission can be greatly improved [Fig. 3(b)]. These experimental results agree well with the simulations.

**Figure 5:**
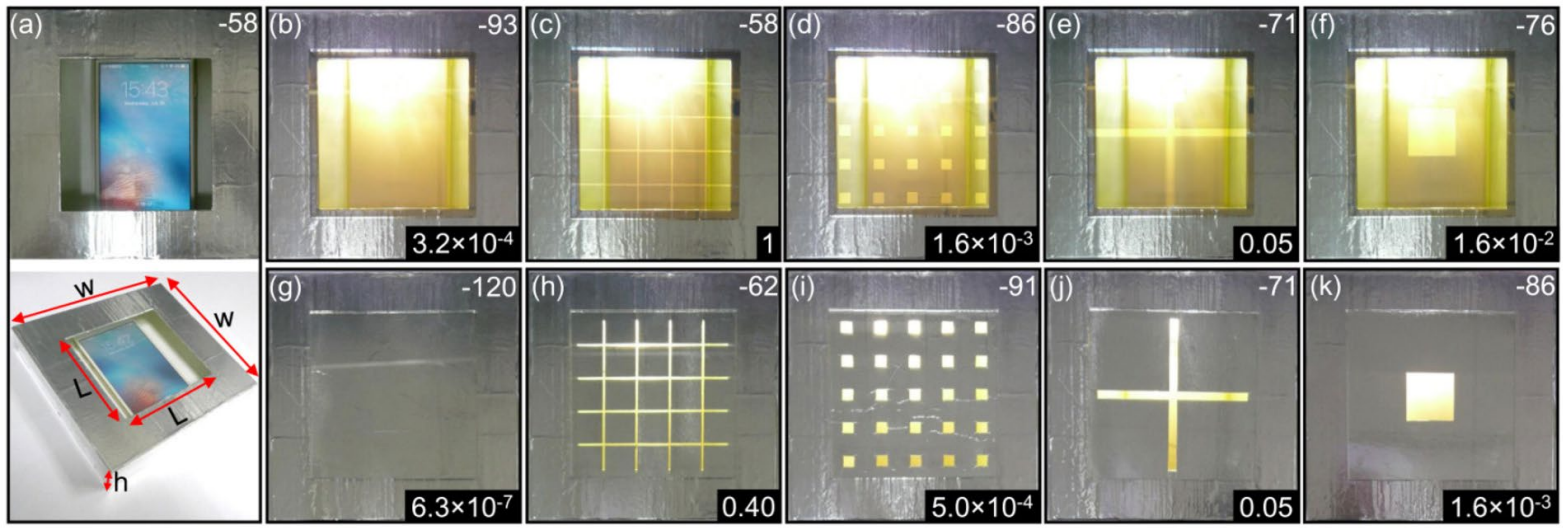
Examination of the signals received by a cell phone placed in a box with different windows. (**a**) The window is open. The top (bottom) panel is the top (3D) view of the box. (**b**) The window is covered with a piece of 1 mm-thick glass coated by 3-nm-thick TiO_2_, 1-nm-thick Au, 12-nm-thick Ag, 1-nm-thick Au and 100-nm-thick TiO_2_. (**c**–**f**) The same as (**b**) but with air in the metallic coatings, where the filling ratio of air is 0.1 (*i.e*. the total area of air is fixed to be 8.1 cm^2^). The air [metal] parts are connected in (**c**,**e**) [(**d**,**f**)]. The period *a* = 2 cm in (**c**,**d**). (**g**–**k**) The same as (**b**–**f**), but with the glass coated by 16 *μ*m-thick Al foil. The box is fabricated with PLA by using 3D printing and covered by 16 *μ*m-thick Al foil. The box has a wall thickness of 1 mm, *w* = 15 cm, *h* = 3 cm, and *L* = 9 cm. The numbers in the top right corners of the photos are the received power in unit of dBm, while the bottom ones are transmissions through the windows.

We also prepared ten samples that are uniform and patterned Ag films and Al foils covered on glass with size of 10 cm × 10 cm [see Fig. 5(b–k)]. These samples are mounted separately on a 9 cm × 9 cm window of a 15 cm × 15 cm × 3 cm box, which is made of polylatic acid (PLA) and then covered by Al foil [see Fig. 5(a)]. Inside the box is placed a cell phone which supports 4 G network. Using the engineering mode of the cell phone, we can obtain the intensity of received signals by the cell phone and thus the transmission of samples.

When a uniform Ag film (Al foil) is applied, the transmission is found to be as low as 3.2 × 10^−4^ (6.3 × 10^−7^). This transmission can be changed if the metallic film is perforated by periodic holes or air grids. Here, the total area of air is fixed to be 8.1 cm^2^, which is 10% of the window area (81 cm^2^). It is found that when Ag film (Al foil) is perforated by air grids with period *a* = 2 cm and 9 cm, the transmission can be increased to 1 (0.40) and 0.05 (0.05) respectively. In contrast, if Ag film (Al foil) is perforated by periodic holes with period *a* = 2 cm and 9 cm, low transmission of 1.6 × 10^−3^ (5 × 10^−4^) and 1.6 × 10^−2^ (1.6 × 10^−3^) can be observed, respectively. The experimental results agree well with above theoretical predictions.

## Discussion

We have shown that the introduction of metallic metasurfaces can greatly improve the transmission of microwaves through low-e materials. By investigating the impact of certain geometry patterns, namely air-connected and metal-connected structures, and the periodicities on transmission, we found that the transmission is sensible to the structure and period of metasurface. Air-connected structures with period of *a* < *λ*/4 and air filling ratio of 10% are recommended because of the high transmission in 4G band. Both simulations and experiments were also conducted to support the conclusion. The use of metasurface is highly beneficial in the low-e materials. The advantages of easy to process, low cost, large-area fabrication and design versatility make it a promising candidate to solve the indoor outdoor communication problem.

## Methods

### Sample preparations

In order to fabricate low-e glass with patterned coating layers, masking tape with a designed pattern is first covered on a piece of glass. By using electric beam evaporation deposition, 3-nm-thick TiO_2_, 1-nm-thick Au, 12-nm-thick Ag, 1-nm-thick Au and 100-nm-thick TiO_2_ are then covered on top of the glass. Similarly, to prepare samples with patterned Al foils, Al foil is first adhered on the top of glass by using double-sided tape, and then patterned by using a utility knife.

### Infrared absorption/emissivity measurements

The infrared absorption spectrum is measured by a Bruker Hyperion-1000 Fourier transform infrared spectrometer (FTIR).

### Microwave transmission spectra measurements

A 1 m × 1 m aluminum board is opened with a 12 cm × 12 cm square aperture in the center. A 14 cm × 14 cm sample is then mounted on the aperture. A horn antenna (750 MHz–18 GHz) is located on the left/right side of the aperture, which is connected with Agilent N5230C vector network analyzer (VNA) to emit/receive microwaves. By comparing the received signals with and without a sample on the aperture, the transmission through the sample can be obtained.

### Characterizations with a cell phone

A 15 cm × 15 cm × 3 cm box is fabricated with polylatic acid (PLA) by using 3D printing. A 9 cm × 9 cm window exists on the top of the box, which can be covered by a 10 cm × 10 cm sample such as low-e glass. There is a 0.5 cm × 1 cm aperture on the bottom of the box, which is covered by a metallic window film (*i.e*. a Polyethylene terephthalate (PET) sheet with a 18 nm-thick-Ag film inside) which is semi-transparent for visible light and opaque for RF signals. To block RF signals, all the PLA surfaces of the box are covered by Al foils.

A cell phone supporting 4 G network is placed inside the box. By using the engineering mode, the intensity of received signal *I* can be shown on the screen of cell phone, where *I* = 10 lg(*P*/*P*_0_), *P* is the power received, and *P*_0_ = 1 mW. This intensity *I* can be observed through the aperture on the bottom of the box.

When the window of the box is open, the intensity of received signal is *I*_0_ (−57 dBm in our experiments). If a metallic surface is covered on the window, the intensity of received signal becomes *I*′, corresponding to transmission ${T}_{exp}={10}^{({I}^{^{\prime} }-{I}_{0}\mathrm{)/10}}$. Here, the metallic surfaces of samples are close to the box to achieve better testing effects.
